# Dengue activates mTORC2 signaling to counteract apoptosis and maximize viral replication

**DOI:** 10.3389/fcimb.2022.979996

**Published:** 2022-09-12

**Authors:** Christoph C. Carter, Fred D. Mast, Jean Paul Olivier, Natasha M. Bourgeois, Alexis Kaushansky, John D. Aitchison

**Affiliations:** ^1^ Center for Infectious Disease Research, Seattle, WA, United States; ^2^ Division of Allergy and Infectious Diseases, Department of Medicine, University of Washington, Seattle, WA, United States; ^3^ Center for Global Infectious Disease Research, Seattle Children’s Research Institute, Seattle, WA, United States; ^4^ Department of Global Health, University of Washington, Seattle, WA, United States; ^5^ Department of Pediatrics, University of Washington, Seattle, WA, United States; ^6^ Department of Biochemistry, University of Washington, Seattle, WA, United States

**Keywords:** mechanistic target of rapamycin (mTOR), mechanistic target of rapamycin complex 2 (mTORC2), apoptosis, non-structural protein 5, flavivirus, pathogenesis, viral replication, dengue virus (DENV)

## Abstract

The mechanistic target of rapamycin (mTOR) functions in two distinct complexes: mTORC1, and mTORC2. mTORC1 has been implicated in the pathogenesis of flaviviruses including dengue, where it contributes to the establishment of a pro-viral autophagic state. Activation of mTORC2 occurs upon infection with some viruses, but its functional role in viral pathogenesis remains poorly understood. In this study, we explore the consequences of a physical protein-protein interaction between dengue non-structural protein 5 (NS5) and host cell mTOR proteins during infection. Using shRNA to differentially target mTORC1 and mTORC2 complexes, we show that mTORC2 is required for optimal dengue replication. Furthermore, we show that mTORC2 is activated during viral replication, and that mTORC2 counteracts virus-induced apoptosis, promoting the survival of infected cells. This work reveals a novel mechanism by which the dengue flavivirus can promote cell survival to maximize viral replication.

## Introduction

Mechanistic target of rapamycin (mTOR) is a ubiquitous, essential serine/threonine kinase that functions in several key aspects of cell biology [reviewed in (Laplante and Sabatini, 2009; Battaglioni et al., 2022; Simcox and Lamming, 2022)]. mTOR exerts its actions as a component of two distinct complexes, mTORC1 and mTORC2. mTORC1 is composed of mTOR, raptor, and mLST8, with PRAS40 and deptor also being present in some cell types (Simcox and Lamming, 2022). mTORC1 functions as a master regulator of anabolic/catabolic homeostasis. In conditions of nutritional abundance, mTOR phosphorylates p70 ribosomal protein S6K (S6K) and eukaryotic initiation factor 4E binding protein 1 (4E-BP1), leading to increased protein synthesis. In conditions of nutrient scarcity mTORC1 is inactivated, stimulating autophagy, which allows for the recycling and turnover of cellular organelles and protein complexes (Laplante and Sabatini, 2009).

mTORC2 is composed of mTOR, rictor, mLST8, and SIN1 (Laplante and Sabatini, 2009; Battaglioni et al., 2022; Simcox and Lamming, 2022). mTORC2 has distinct roles from mTORC1 in cellular physiology, but these roles are less well understood than those of mTORC1. mTORC2 promotes cell survival and proliferation through phosphorylation of AKT at ser473 (Laplante and Sabatini, 2012). It is also known to play a role in the maintenance of the actin cytoskeleton, and when inactivated results in morphological abnormalities in some cell lines (Jacinto et al., 2004; Sarbassov et al., 2004). It has been suggested that mTORC2 may modulate translational machinery due to its association with ribosomes; however, the ramifications of this interaction are not well understood (Zinzalla et al., 2011). Although mTORC1 is the canonical regulator of autophagy, mTORC2 has been implicated in the regulation of specific autophagic processes such as chaperone-mediated autophagy and mTORC1-independent autophagy (Arias et al., 2015; Lampada et al., 2017).

Given the important role of mTORC1 in regulating cellular metabolism, it is not surprising that several viruses have evolved mechanisms to modulate mTORC1 signaling (Buchkovich et al., 2008; Le Sage et al., 2016). Numerous viruses have been shown to manipulate mTORC1 activity during infection; some viruses activate mTORC1 to maintain cellular anabolic machinery, whereas others suppress mTORC1 activity to favor cap-independent viral protein synthesis (Buchkovich et al., 2008; Le Sage et al., 2016). Accordingly, mTOR is actively being explored as a potential host based anti-viral therapeutic target (Maiese, 2020).

In the case of dengue, virus-induced modulation of mTORC1 has been suspected due to the importance of autophagy in dengue infection (Lee et al., 2008; Heaton and Randall, 2010; Lee et al., 2013; Metz et al., 2015), although this interaction of the virus with mTORC1 has not been comprehensively investigated. mTORC signaling is also dysregulated in dengue infection of megakaryocytes (Lahon et al., 2021). The importance of mTORC1 in dengue replication is supported by studies demonstrating increased viral replication in the response to pharmacologic mTOR inhibition and a recent study implicating mTORC1 in the dengue-induced activation of lipophagy (Mateo et al., 2013; Jordan and Randall, 2017). In contrast to mTORC1, potential roles for mTORC2 in virus-host interaction are comparatively poorly understood. Activation of mTORC2 has been documented in human cytomegalovirus, West Nile and influenza infection (Kudchodkar et al., 2006; Shives et al., 2014; Kuss-Duerkop et al., 2017), but the functional role of mTORC2 in these infections remains unknown. Furthermore, to our knowledge no role for mTORC2 has been described in dengue infection.

Here, we describe a role for mTORC2 in promoting cell survival during dengue infection. We find that the dengue non-structural protein 5 (NS5) interacts with mTORC1 and mTORC2 complexes, and that dengue infection leads to the activation of mTORC2 signaling. We report that inactivation of mTORC2 signaling leads to a decrease in viral replication and an increase in virus-induced apoptosis and cell death. These findings suggest a mechanism by which dengue counteracts apoptosis to maintain cell survival and maximize viral replication.

## Results

### Dengue NS5 protein interacts with mTORC1 and mTORC2

**Figure 1:**
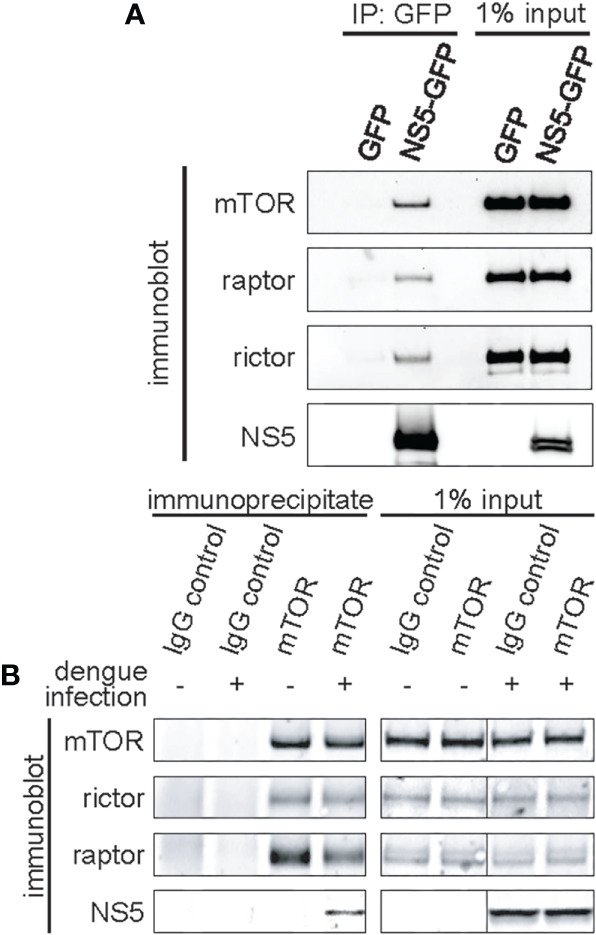
Dengue NS5 interacts with mTORC1 and mTORC2. **(A)**, Immunoprecipitation of NS5-GFP fusion protein identifies mTORC1 and mTORC2 interacting proteins. 293FT cells were transfected with expression plasmids encoding GFP or NS5-GFP fusion protein, and NS5-GFP was affinity captured from the cell lysate using anti-GFP nanobodies. 50% of the eluates or 1% of the input lysate were then assayed by western blot analysis with the indicated antibodies. **(B)**, mTOR interacts with NS5 during dengue infection. HepG2 cells were infected with dengue virus, serotype 2, at an MOI of 4. mTOR was immunoenriched from the lysate using anti-mTOR antibodies. Separately, nonspecific IgG was used as an immunoprecipitation control. 50% of the eluates or 1% of the input lysate were then assayed by western blot analysis with the indicated antibodies.

Quantitative proteomics [I-DIRT; Isotopic Differentiation of Interactions as Random or Targeted (Tackett et al., 2005)], designed to identify *bona fide* dengue-host protein-protein interactions, defined a high-confidence protein interaction network including a predicted interaction between the dengue NS5 protein and mTOR (Carpp et al., 2014). To validate and further study this interaction, we performed co-immunoprecipitation experiments using exogenously expressed NS5. GFP-tagged NS5 or GFP alone was transfected into 293FT cells, leading to modest expression of the fusion protein in the cytosol with nuclear accumulation (**Supplemental Figure 1**), as has been previously reported (Kumar et al., 2013). Protein complexes were then affinity purified using GFP-specific nanobodies (Fridy et al., 2014). Subsequent western blotting of affinity purified NS5 complexes revealed mTOR, raptor, and rictor proteins, demonstrating that NS5 interacts with both mTORC1 and mTORC2 (**Figure 1A**).

We also assayed for the NS5-mTORC interaction in dengue-infected HepG2 hepatoma cells (**Figure 1B**). HepG2 hepatoma cells were used due to the hepatotropic nature of dengue, and the extensive previous research of dengue with this cell line, including its replication, and its roles in modulating host processes such as apoptosis, autophagy and ER stress (Suksanpaisan et al., 2007; Thepparit et al., 2013; Carpp et al., 2014; Jordan and Randall, 2017). mTOR was immunopurified and the eluate probed for NS5. As a control, we also probed for rictor and raptor (**Figure 1B**). As expected, NS5 was immunopurified in the mTOR pulldown in dengue-infected cells, but absent in the IgG immunoprecipitation control. Intriguingly, less raptor protein associated mTOR in dengue-infected cells, whereas rictor was not impacted (**Figure 1B**). These experiments confirm the previous I-DIRT results (Tackett et al., 2005) and establish the sufficiency of NS5 alone for interacting with mTORC1 and mTORC2, i.e. not requiring the presence of other viral proteins, such as NS3, to initiate and/or stabilize the interaction.

### mTORC2 is activated during dengue replication and is required for efficient viral replication

**Figure 2:**
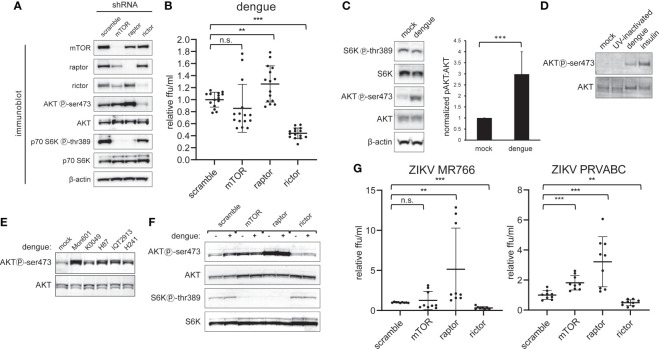
Dengue infection activates mTORC2 signaling and is required for maximal viral replication. *A*, Selective inactivation of mTORC1 and mTORC2 in HepG2 cells. Cells were transduced with lentivirus encoding shRNA directed against mTOR protein, Raptor, or Rictor, or with a non-specific scramble sequence, and then selected with puromycin. Lysates were analyzed by western blot with the indicated antibodies. *B*, mTORC2 inactivation diminishes dengue replication. Cells were transduced with lentiviral shRNA vectors as in **(A)** and were then infected with dengue MON601 at a MOI of 1. 24 h later cell culture supernatants were collected and titrated on Vero cells. The data represent scramble-normalized values from 4 independent experiments. **(C)**, mTORC2 is activated during dengue infection. HepG2 cells were infected with dengue at MOI of 4. Cells were collected at 36 h post-infection and analyzed by western blot with the indicated antibodies. The bar graph shows the ratio of phospho-AKT to total AKT band intensity averaged from 7 independent experiments. **(D)**, Competent dengue virus infection is required for AKT activation. HepG2 cells were infected with dengue at a MOI of 1, or with UV-inactivated virus. Mock and insulin treatment were used as a negative and positive control for AKT activation, respectively. Lysates were analyzed by western blot with the indicated antibodies. **(E)**, mTORC2 activation is conserved across dengue serotypes. Experiments were performed as in **(C)** using Huh7 cells infected with DENV-2 MON601, DENV-2 K0049, DENV-3 H87, DENV-2 IQT2913, or DENV-4 H241. **(F)**, Rictor knockdown abrogates mTORC2 activation by dengue. Cells were transduced with shRNA encoding lentivirus as in **(A)** and were infected with dengue at MOI of 4. Cells were collected at 36 hpi and were analyzed by western blot with the indicated antibodies. **(G)** mTORC2 abrogation negatively impacts Zika virus (ZIKV) replication. Experiments were performed as in **(B)** but with ZIKV MR788 and ZIKV PRVABC. Error bars are one standard deviation. p values are derived from 2-tailed Student’s t test; n.s., not significant; ** denotes p < 0.01, and *** denotes p < 0.001.

To investigate the impact of NS5-mTOR interactions on dengue viral infection, and to explore the role of NS5 interacting with mTORC1 and mTORC2, the expression of each complex was silenced using lentivirus-delivered shRNA targeting mTOR, raptor (a component of mTORC1) or rictor (a component of mTORC2) or, as a control, a nonspecific scrambled oligo-sequence (Sarbassov et al., 2005). Transduction of the corresponding shRNA resulted in substantially decreased protein abundance of mTOR, Raptor or Rictor (**Figure 2A**). To assess whether raptor and rictor knockdown disrupted the signaling activity of mTORC1 and mTORC2, we examined the phosphorylation status of well-characterized downstream targets (p70 S6K thr389 for mTORC1 and AKT ser473 for mTORC2) (Burnett et al., 1998; Sarbassov et al., 2005). Knockdown of raptor led to diminished mTORC1 signaling as evidenced by decreased S6K thr389 phosphorylation, and knockdown of rictor led to diminished mTORC2 signaling as evidenced by decreased AKT ser473 phosphorylation (**Figure 2A**). We observed that depletion of mTORC1 led to a modest reciprocal activation of mTORC2 activity, consistent with prior reports suggesting that mTORC1 represses mTORC2 signaling (Julien et al., 2010) (**Figure 2A**). The converse did not appear to be the case, as depletion of mTORC2 did not lead to increased phosphorylation of S6K by mTORC1 (**Figure 2A**). Furthermore, mTOR knockdown diminished levels of raptor and rictor, but knockdown of neither raptor nor rictor influenced levels of each other (**Figure 2A**).

We next assessed the effect of reduced mTOR, raptor (mTORC1), or rictor (mTORC2) protein levels on dengue serotype 2 (DENV2) replication by infecting respective knockdown cells and quantifying the amount of infectious virus released (**Figure 2B**). Interestingly, rictor knockdown led to a substantial decrease in the amount of viral replication, while raptor knockdown led to a small but significant increase in replication. No significant effect on viral replication was observed when mTOR protein was knocked down (**Figure 2B**).

Having observed a decrease in viral replication with mTORC2 inactivation, we next asked whether dengue infection affected the activity of mTORC2 (**Figure 2C**). HepG2 cells were infected with DENV-2 and lysates were probed for the phosphorylation status of the downstream targets AKT and S6K by western blot. We observed an increase in mTORC2-specific phosphorylation at AKT ser473 in infected cells compared to the mock treatment, with the ratio of ser473 phosphorylated AKT to total AKT significantly increased in infected cells (**Figure 2C**, p < 0.001). In contrast, AKT ser473 phosphorylation was not increased when cells were treated with UV-inactivated virus (**Figure 2D**).

To assess the breadth of AKT activation resulting from dengue infection, Huh7 cells were separately infected with three isolates of DENV-2 (MON601, K0049 and IQT2913) or an isolate of DENV-3 (H87), or DENV-4 (H241). In all cases, we observed an increase in the phosphorylation of AKT ser473 (**Figure 2E**). The subtle variation in degree of AKT phosphorylation may correspond to variable infectivity across the isolates or strain specific differences in the degree of activation; nonetheless, all strains activated AKT.

Abrogation of the increased AKT ser473 phosphorylation was observed in cells depleted for rictor, but not mTOR or raptor. As in **Figure 2A**, knockdown of mTOR protein or raptor led to increased mTORC2 activity, which was not further increased by dengue infection (**Figure 2F**). These experiments demonstrate that mTORC2 is required for dengue-induced AKT ser473 phosphorylation.

To evaluate if mTORC2’s role in dengue virus replication is conserved in other flaviviruses, we infected HepG2 cells depleted for mTOR, raptor, or rictor with two separate isolates of the Zika virus (ZIKV MR766 and PRVABC59). In each case, increased viral replication was observed in cells depleted for raptor while decreased viral replication was observed in cells depleted for rictor (**Figure 2G**). These data suggest that the role of mTORC2 on infection is conserved in the related flavivirus, Zika virus.

### mTORC2 inhibition does not affect cellular morphology, growth rates or dengue-induced LC3-II accumulation

**Figure 3:**
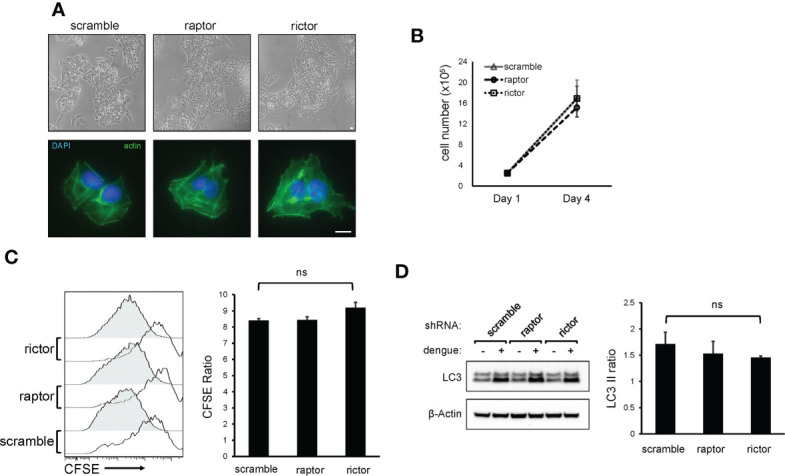
mTORC2 inactivation does not alter HepG2 cell morphology, cytoskeletal architecture, growth rate, or LC3 lipidation in response to dengue infection. **(A)**, HepG2 cells were transduced with lentiviral vectors encoding shRNA directed against mTOR protein, Raptor or Rictor, or with a non-specific scramble sequence and were selected with puromycin. Cells were imaged by light microscopy (top panels) or stained with fluorophore-labeled phalloidin and imaged with fluorescence microscopy (bottom panels). Bar = 100 µm **(B)**, Cells were transduced with shRNA lentiviral vectors as in **(A)** and were plated at equal densities. Cells were then counted using an automated cell counter 4 days later. Data represent cell counts from 4 separate cultures. **(C)**, Cells were transduced with shRNA lentiviral vectors as in **(A)** and were plated at equal densities. Cells were then loaded with CFSE and analyzed by flow cytometry 24 h (open histograms) and 96 h (shaded histograms) later. Bar graph represents the ratio of CFSE mean fluorescence intensity between the 24 and 96 h timepoint, averaged from 4 separate cultures. **(D)**, mTORC2 inhibition does not alter dengue-mediate LC3-II lipidation. Cells were transduced with shRNA lentiviral vectors as in **(A)** and were infected with dengue at MOI 4. Cells were harvested at 36 h post-infection and lysates were analyzed by western blot with the indicated antibodies. The bar graph shows the ratio of LC3-II band intensity between dengue infected and mock treated cells from 3 independent experiments. Error bars are one standard deviation. p values are derived from 2-tailed Student’s t test; n.s., not significant.

The increase in mTORC2 activity during dengue infection and the requirement for mTORC2 activity for maximal viral replication led us to investigate potential physiological functions of mTORC2 that are exploited by the virus. Because mTOR has also been shown to play a role in the maintenance of cell morphology by modulating ion channel activity and altering dynamics of the actin cytoskeleton (Jacinto et al., 2004; Sarbassov et al., 2004), we examined the morphology of mTORC2-depleted cells and the distribution of their actin cytoskeletons by microscopy. Cells treated with shRNA targeting rictor showed normal cellular morphology, normal cell spreading and a morphologically normal actin cytoskeleton (**Figure 3A**), suggesting that mTORC2 inactivation was not causing derangements in cell architecture in this setting. mTORC2 has been shown to play a role in regulating cell proliferation (Oh and Jacinto, 2011), so we considered the possibility that our findings could be explained by altered cell growth rates. We assessed cell growth rates in knockdown cells by measuring cell counts and CFSE dilution. No significant differences in growth rates were observed upon comparison of mTORC2 knockdown cells with control cells (**Figures 3B, C**).

Although autophagy is canonically regulated by mTORC1, we also considered the possibility that inactivation of mTORC2 suppresses dengue replication by altering autophagy through indirect regulatory effects on mTORC1. While the effect that dengue has on the movement of cargo through the canonical autophagy pathway is unclear, it is well known that dengue induces the accumulation of autophagosomes, characterized by accumulation of lipidated LC3 protein (LC3-II) (Lee et al., 2008; Heaton and Randall, 2010; Metz et al., 2015; Jordan and Randall, 2017). To assess effects of mTORC2 inhibition on the pro-autophagic activity of dengue, we measured LC3-II isoform levels in dengue-infected knockdown cells and observed similar degrees of dengue-induced LC3-II accumulation (**Figure 3D**), suggesting that mTORC2 inactivation does not block the effects of dengue on autophagy.

### mTORC2 inactivation causes increased dengue-mediated apoptosis and cell death

**Figure 4:**
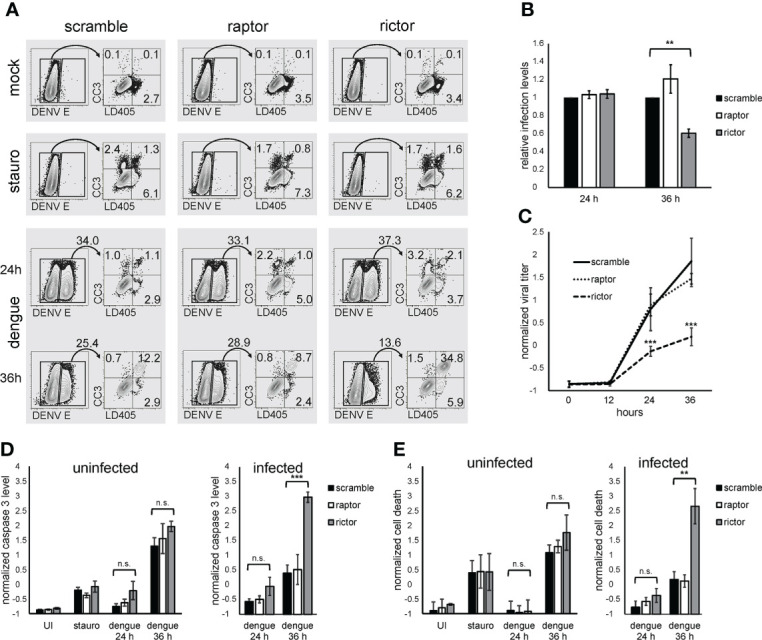
mTORC2 inhibition leads to increased dengue-induced apoptosis and cell death. **(A)**, HepG2 cells were infected with dengue at MOI of 4 for 24 or 36 h, mock treated, or treated with 5 µM staurosporine overnight. Cells were then collected and stained with a cell-impermeable amine reactive dye (LD405), followed by fixation, permeabilization, and staining with anti-activated caspase 3 and anti-flavivirus E protein antibodies. Infected (E-positive) cells were gated and activated caspase 3 (CC3) and LD405 expression were analyzed within that population. Numbers represent population frequencies in each gate/quadrant. **(B)**, Cells were treated as in **(A)**, and the percent of infected (E-positive) cells was calculated. The bar graph shows the average values for 3 independent experiments, each normalized to the scramble condition. **(C)**, Cells were treated as in **(A)**, and the amount of infectious virus present in the supernatant was quantified at the indicated timepoints. The bar graph represents Z-normalized average values from 3 independent experiments. **(D)**, Cells were treated as in **(A)**, and the percent of infected cells positive for caspase 3 was calculated. The bar graph represents *Z*-normalized values from 3 independent experiments. **(E)**, Cells were treated as in **(A)**, and the percent of infected cells positive for LD405 was calculated. The bar graph represents *Z*-normalized values from 3 independent experiments. p values are derived from 2-tailed Student’s t test; n.s., not significant; ** denotes p < 0.01, and *** denotes p < 0.001.

mTORC2 could function as a pro-survival factor in infected cells, given the known role for mTORC2 in regulating cell survival (Oh and Jacinto, 2011; Lamming et al., 2014; Zou et al., 2016). Rictor knockdown or scramble control cells were infected with dengue for 24 or 36 h and, as a positive control for apoptosis, treated the cells with staurosporine. We then asked whether cell death, or apoptosis specifically, was altered in the context of mTORC2 inhibition by measuring activated caspase 3 expression and cell viability within infected and uninfected cells using flow cytometry. At 24 hours post-infection (hpi), we observed a similar frequency of infected cells when comparing control cells to rictor or raptor knockdown cells, arguing against an early block in viral replication in mTORC2-deficient cells. However, at 36 hpi, rictor knockdown was associated with a significant decrease in the proportion of infected cells (**Figures 4A, B**). In contrast, raptor knockdown cells exhibited a non-significant increase in infection. Rictor knockdown cells also produced less infectious virus at 24 and 36 hpi, whereas raptor knockdown cells produced virus at levels indistinguishable from that produced by the scramble control cells (**Figure 4C**). Assessment of activated caspase 3 expression and cell viability at 24 h revealed a small increase in apoptosis and cell death in rictor-deficient cells, which were not statistically significant (**Figures 4A, D, E**). However, by 36 hpi, a marked increase in apoptosis and cell death was observed in infected rictor knockdown cells, both of which were highly significant (4*A*, *D*, and *E*, p < 0.001 for activated caspase 3 level, p < 0.01 for cell viability). Interestingly, in contrast to what we observed in dengue infected cells, staurosporine treatment increased cell death and apoptosis at similar levels in the scramble control, rictor, and raptor knockdown cells, and there were no statistically significant differences in the uninfected populations (**Figures 4A, D, E**). This suggests that the increased apoptosis observed in dengue-infected cells was specific for virus-induced apoptosis in the rictor knockdown cells and not a general property of all pro-apoptosis stimuli. Thus, dengue-infected cells harbor a specific sensitivity to mTORC2 inhibition.

## Discussion

In this study, we describe a role for host mTORC2 in dengue replication, demonstrating that dengue can interact with cellular mTOR signaling, activating mTORC2 and, as a result, promoting cell survival and efficient viral replication. Specifically, the data we present here suggest that mTORC2 plays a role in supporting viral production by counteracting virus-induced apoptosis of the host cell. The induction of apoptosis in dengue infection has been well-described, occurring in several cell types both *in vitro* and *in vivo*, including endothelial cells, dendritic cells, and hepatocytes (Limonta et al., 2007; Torrentes-Carvalho et al., 2009; Martins Sde et al., 2012; Lin et al., 2014). The finding of apoptotic cells in human autopsy specimens from severe dengue cases has led to speculation that apoptosis contributes to pathogenesis in these cases (Limonta et al., 2007). While apoptosis may contribute to tissue damage and pathogenesis from the host perspective, it is also an important mechanism for host control of viral infection, triggering cell death before infectious progeny can be released (Orzalli and Kagan, 2017) and shaping the subsequent immune response.

Since uncontrolled apoptosis would be detrimental to viral replication, it is not surprising that dengue has evolved a mechanism to attenuate the induction of apoptosis in infected cells. In the present study, we found that disrupting mTORC2 signaling in infected cells leads to an increased frequency of apoptosis in cell death, and the induction of apoptosis corresponds to a decrease in the release of viral progeny from infected cells. Interestingly, the susceptibility of mTORC2-deficient cells to apoptosis was specific for dengue infection in our experiments, as neither baseline apoptosis nor apoptosis in response to staurosporine treatment increased over control upon depletion of mTORC2. Furthermore, we found that dengue infection triggers the activation of mTORC2, which likely represents a viral adaptation to maintain cell survival during infection. While this strategy has not been described in other viral infections, it has been reported as mechanism for cancer cell survival and metastasis in several malignancies (Kim et al., 2017). The finding that mTORC2 is a regulator of cell death during dengue infection opens the possibility of host targeted interventions, including synthetic lethal strategies, that serve to tune apoptotic responses in infected cells to limit pathogenesis or viral spread and/or to amplify protective immune responses (Mast et al., 2020).

**Figure 5:**
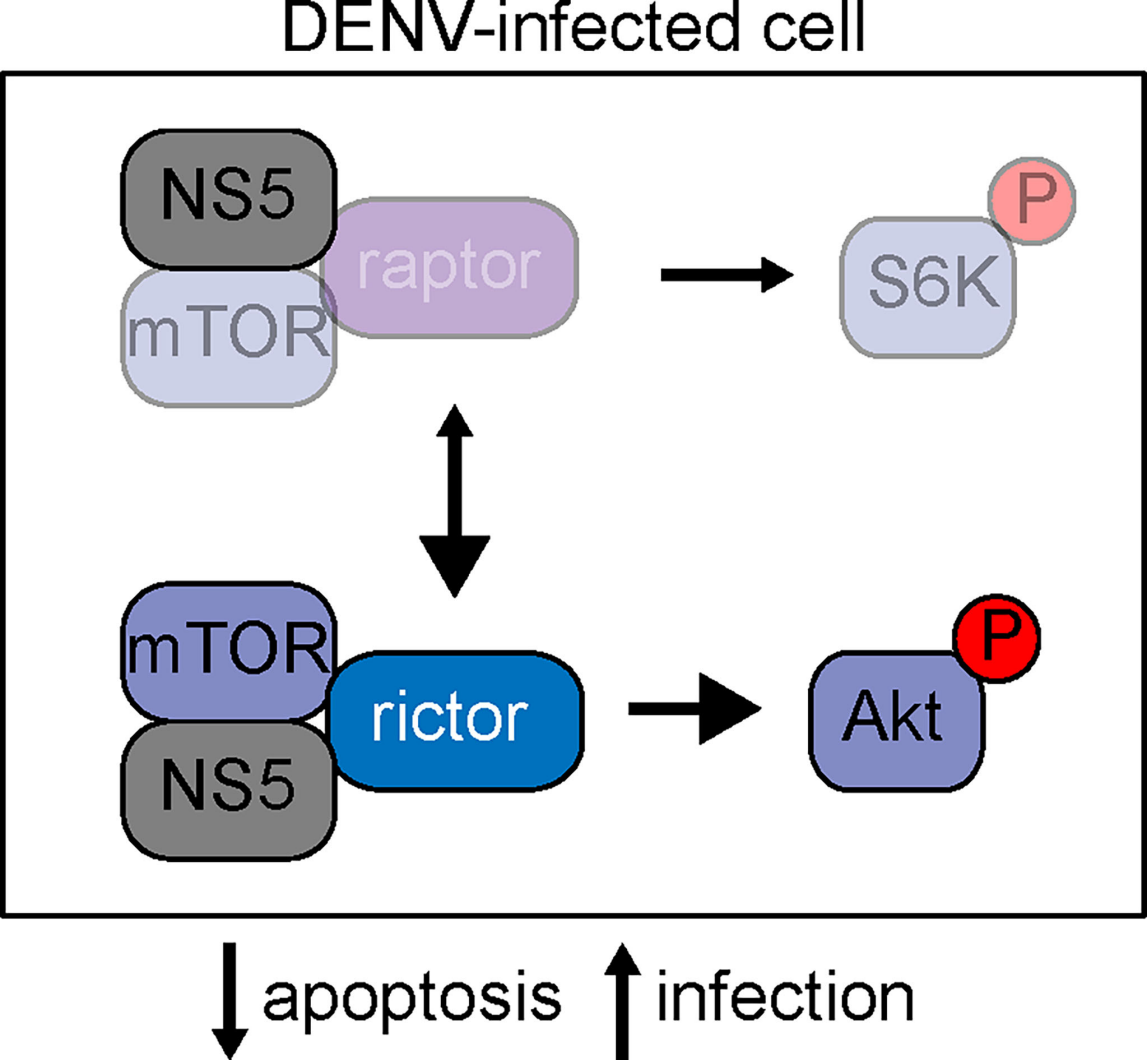
Schematic of mTOR signaling in dengue infection. At resting state, mTOR can participate in two well-characterized complexes, mTORC1 and mTORC2. mTORC1 is characterized by the binding of mTOR to raptor, whereas mTORC2 requires mTOR binding to rictor. Our data is consistent with a model where NS5 binds mTOR, and this binding stabilizes the mTORC2 complex, leading to elevated signaling through phosphorylated Akt and subsequent protection against cellular apoptosis to support viral replication.

Dengue NS5 protein binds to mTOR, suggesting that the viral protein modulates mTOR signaling during infection. NS5 appears to bind both mTORC1 and mTORC2, evidenced by the co-immunoprecipitation of raptor and rictor with NS5 (Carpp et al., 2014). Our data are consistent with a model where this binding event stabilizes the mTORC2 complex, amplifies signaling through Akt, boosts cell survival, and facilitates optimal viral propagation (**Figure 5**). The detailed molecular consequences of the NS5-mTOR interaction remain to be investigated. One possibility is that NS5 binds to mTORC2 and directly facilitates activation of the complex or acts as an adaptor protein to stabilize interactions with downstream targets of the complex. Given that there is crosstalk between mTORC1 and mTORC2 signaling (Tyakht et al., 2014), it is also possible that interaction of NS5 with mTORC1 could activate mTORC2, either *via* de-repression of mTORC2 or through alterations in mTOR protein stoichiometry.

The findings we present here also highlight the role of proteomic approaches in understanding virus-host interactions. Much of the recent focus in dengue research has utilized high-throughput genetic screens employing approaches such as RNAi and CRISPR (Sessions et al., 2009; Marceau et al., 2016; Savidis et al., 2016). While these genetic screens have identified host dependency factors, they rely on measuring viral gene expression or cell survival as the readout for infection resistance, and they are likely biased toward host factors involved in the early stages of infection such as viral entry and gene expression as opposed to host factors needed for late infection events such as viral release, maturation, and host cell survival. For this reason, it is perhaps not surprising that components of the autophagy machinery and mTOR signaling have not been consistently identified in these screens, despite the roles of these host factors reported here and by others (Mateo et al., 2013; Jordan and Randall, 2017). Moreover, genetic screens stop short of identifying specific molecular interactions between virus and host that mediate regulatory processes, which can be elucidated when interactions between viral and host proteins are interrogated *via* proteomics. Nonetheless, it is notable that rictor inhibition was associated with decreased relative infection in 2 RNAi libraries previously reported, although that finding did not meet the authors’ criteria for further validation (Savidis et al., 2016). In contrast to genetic screens, proteomics methods can identify host interactions at all stages of the viral life cycle, and subsequent validation can identify important host interactions. When combined with quantitative approaches to distinguish high-probability interactors from non-specific binding, proteomics approaches can identify host factors with high validity (Carpp et al., 2014).

The observation that mTORC2 is required for efficient dengue replication raises the possibility of mTOR as a target for host-directed antiviral therapeutic development. mTOR is a highly “druggable” target, and intense interest in the mTOR pathways in oncology and neuropathology fields has spurred the development of multiple new small molecule inhibitors with high specificity for mTOR (Xie et al., 2016; Zou et al., 2016). In the solid organ transplant infectious disease field, data have begun to emerge that certain mTOR inhibitors (used as anti-organ rejection medications) may have anti-viral properties, reducing the risk of some viral reactivation syndromes (Pascual et al., 2016). In the case of dengue *per se*, candidate compounds would likely need to have mTORC2 specificity, since mTORC1 inhibition appears to enhance viral replication. While most newer generation mTOR inhibitors have dual mTORC1/C2 specificity, a recently developed compound CID613034 has been described that specifically inhibits mTORC2, demonstrating the feasibility of specific mTORC2 inhibition (Benavides-Serrato et al., 2017). Since multiple biochemical steps occur between mTORC2 assembly and the resultant anti-apoptotic outcome, components of the mTORC2 signaling cascade might also provide useful targets for host-based interventions.

The role of mTORC2 signaling in the pathogenesis of other viral infections remains to be determined; however, there is evidence that other viruses including West Nile, influenza and human cytomegalovirus may also stimulate mTORC2 signaling (Kudchodkar et al., 2006; Shives et al., 2014; Kuss-Duerkop et al., 2017). Whether mTORC2 is important for viral replication and serves as an anti-apoptotic mechanism in those viruses remains to be determined, but it is possible that mTORC2 modulation is a common mechanism used by several viruses to counteract the host’s programmed cell death response. If that is the case, host-directed therapeutic interventions targeting mTORC2 could be active against multiple viral pathogens.

## Materials and methods

### Antibodies and reagents

The following antibodies were obtained from Cell Signaling Technology (Danvers, MA) and used at 1:1000 for western blotting: Raptor clone 24C10, Rictor clone D16H9, phospho-Rictor thr1135 clone D30A6, mTOR 7C10, AKT clone 11E7, phospho-AKT ser473 clone D9E (used at 1:2000), p70 S6K clone 49D7, phospho-p70 S6K thr389 clone 108D2, LC3A/B clone D3U4C, and B-actin clone 13E5. The dengue 2 E protein antibody PA5-32246 was used at 1:10,000 for western blotting (Thermo-Fisher Scientific, Waltham, MA). Secondary detection for western blotting used anti-rabbit HRP antibody diluted 1:10,000 (Amersham ECL, GE Healthcare, Chicago, IL). Polyclonal affinity purified NS3 and NS5 proteins were produced as previously described (Carpp et al., 2014). The pan-flavivirus E antibody 4G2 was prepared from hybridoma supernatants and purified by protein A/G chromatography. For flow cytometry experiments, 4G2 was conjugated to FITC according to the manufacturer’s instructions (FluoroTag kit, Sigma-Aldrich, St. Louis, MO). For immunoprecipitation, the dimerized GFP nanobody construct LaG-16–G4S–LaG-2 (green lobster; gift from Michael Rout), mTOR 7C10, and Normal Rabbit IgG (2729S; Cell Signaling Technology, Danvers, MA), were prepared as previously described (Fridy et al., 2014). For microscopy, phalloidin-Alexafluor 488 was obtained from Thermo-Fisher scientific and used according to their instructions. Staurosporine was obtained from Millipore Sigma (Burlington, MA) and was used at 5 µM concentration.

### Plasmids

The lentiviral shRNA plasmids pLKO.1 scramble, Raptor_2, mTOR_2, and Rictor_2 were gifts from David Sabatini (Addgene plasmids 1864, 1858, 1856, and 1854). pMD2.G and psPAX2 were gifts from Didier Trono (Addgene plasmids 12259 and 12260). Dengue NS5-GFP fusion construct was generated by inserting the dengue New Guinea C NS5 coding sequence from pDVW601 (Pryor et al., 2001) into pACGFP1-N1 (Clontech) as previously described (Carpp et al., 2014).

### Cell culture

HEK293FT, HepG2, Huh7 and Vero cells were obtained from the ATCC (Manassas, VA) and cultured at 37°C in 5% CO_2_, in medium composed of MEM supplemented with 10% FBS, 1× non-essential amino acids, 50 units/mL of penicillin and 50 µg/mL of streptomycin. Medium was replenished frequently during experiments to avoid signaling changes caused by nutrient or growth factor depletion. The *Aedes albopictus* derived cell line C6/36 was propagated at 28°C in 5% CO_2_ in medium composed of MEM supplemented with 10% FBS, 1× non-essential amino acids, 50 units/mL of penicillin and 50 µg/mL of streptomycin. The B lymphocyte hybridoma cell line D1-4G2-4-15 was obtained from the ATCC (Manassas, VA), and was maintained in ATCC Hybri-Care Medium supplemented with 10% FBS and 1.5 g/L sodium bicarbonate.

### Transfections

HEK293FT cells were plated to 70-80% density in antibiotic free medium. Transfection complexes were prepared by mixing plasmid DNA with polyethyleneimine (PEI Max 40k, Polysciences Inc., Warrington, PA) in a 1:4 mass ratio. After 15 min incubation at room temperature DNA complexes were added dropwise to the cell cultures.

### Affinity capture

To prepare affinity-capture beads, the dimerized GFP nanobody LaG-16–G4S–LaG-2 (green lobster), mTOR IgG, and non-specific IgG were covalently linked to magnetic beads (Dynabeads M-270 epoxy Thermo Fisher Scientific) as previously described (Cristea and Chait, 2011). Briefly, 5 μg of nanobody were used per 1 mg of Dynabeads, with conjugations carried out in 0.1 M sodium phosphate buffer and 1 M ammonium sulfate, with an 18- to 20-h incubation at 30°C. Beads were then washed sequentially with 0.1 M sodium phosphate buffer, 100 mM glycine pH 2.5, 10 mM Tris-HCl pH 8.8, 100 mM triethylamine, 1× PBS (4 times), PBS + 0.5% Triton X-100, and 1× PBS. For affinity capture experiments, cells were harvested 48 h after transfection, or 24 h post DENV infection. Cells were washed with ice cold PBS and then lysed with 1% Triton X-100, 0.5% sodium deoxycholate, 110 mM potassium acetate pH 7.5, 20 mM HEPES, 2 mM MgCl_2_, 25 mM NaCl, and 1× protease/phosphatase inhibitor cocktail (Cell Signaling Technology). Lysates were clarified by centrifugation for 10 min at 13,000 × *g* at 4°C. Affinity capture beads were immediately added to the clarified lysate and incubated for 10 min at room temperature with rotation. Beads were then washed 3× with lysis buffer, and bound protein complexes eluted with 1.1× LDS sample buffer for 10 min at 70°C. For SDS-PAGE and western blot analysis, 10× reducing agent (Thermo-Fisher Scientific) was added and samples were heated for an additional 10 min at 70°C. SDS-PAGE and western blot analysis were performed as described below. Gel staining was performed using Sypro Ruby fluorescent gel stain (Thermo-Fisher Scientific) according to the manufacturer’s instructions.

### shRNA mediated gene silencing

To generate lentiviral vector stocks, shRNA constructs were cotransfected with pMD2.G and psPAX2 into HEK 293T cells. Supernatants were harvested, passed through 0.45 µm filters, layered on 20% sucrose cushions, and centrifuged at 100,000 × *g* for 4 h at 4°C. Lentiviral pellets were resuspended in OptiMEM and stored at -80°C until use. For lentiviral transductions, viral stocks were diluted to the desired concentration with OptiMEM and 0.8 µg/mL polybrene and added to cells. At 48 h post-transduction, cells containing stably integrated constructs were selected using 2 µg/mL puromycin. Experiments were performed on cell lines that were maintained and passaged for no more than 3 weeks before discarding and establishing fresh cell lines.

### Virus and infections

DENV-2 MON601, a molecular clone of DENV-2 New Guinea strain C[46]; DENV-2 K0049, a clone of a southeast Asian isolate; DENV-3 H87, a clone of a Philippines isolate from 1956; DENV-2 IQT2913, a clone of a Peru isolate from 1996; DENV-4 H241, a clone of a Philippines isolate from 1956; ZIKV MR766, a clone of a Ugandan ZIKV isolate from 1947; and ZIKV PRVABC, a clone of a Puerto Rican ZIKV isolate from 2015, were generated by transfection of *in vitro*-transcribed RNA into Vero cells, followed by no more than 5 passages in C6/36 cells. Virus was propagated by interchangeably infecting 80% confluent C6/36 or Vero monolayers with low-passage stock virus at a MOI of 0.01, and harvesting infectious supernatants 5-7 days post-infection. Infectious supernatants were cleared of cellular debris by centrifugation and filtration through 0.2µm PVFD membrane then stored at -80°C until use. Virus stocks and experimental infectious supernatants were titrated using a flow cytometry approach which has been described elsewhere (Lambeth et al., 2005). Briefly, serially diluted virus stocks were used to infect Vero cells in a multi-well plate. Cells were harvested 20-24 h post-infection, fixed and permeabilized, and stained with 4G2-FITC. The percentage of infected cells was then used to calculate the number of fluorescence forming units (FFU) per milliliter of inoculum. To generate inactivated virus, the virus stock was irradiated by ultraviolet light for one hour at room temperature prior to infection. For experimental infections, virus was diluted in OptiMEM to the desired MOI and incubated on cells for 90 min at 37°C. Virus was then removed, the cells washed, and complete growth medium added. To inactivate virus, virus was exposed to UV light for 1 h in a 6-well plate in a biosafety cabinet.

### Western blot analysis

Cells were placed on ice and washed with ice-cold PBS. Cells were then collected and lysed with Triton X-100 lysis buffer (1% Triton X-100, 120 mM NaCl, 1 mM EDTA, 40 mM HEPES pH 7.4, and 1× protease and phosphatase inhibitor cocktail (Cell Signaling Technology)). To prepare whole cell extracts for the immunoprecipitation load control, SDS lysis buffer (2% SDS, 50 mM Tris pH 7.4, 5% glycerol, 5 mM EDTA, 1 mM NaF, 1 mM DTT, and 1× phosphatase & protease inhibitor cocktail) was used instead of Triton X-100 lysis buffer. Protein concentration was determined using BCA assay and a BSA standard curve, and equivalent amounts of protein were mixed with 4× LDS sample buffer and 10× reducing agent (Thermo-Fisher Scientific), followed by denaturation at 70°C for 10 min. Proteins were then resolved on 4-12% Bis-Tris gels (for lower molecular weight proteins) or 3-8% Tris-Acetate gels (for higher molecular weight proteins) (NuPage, Thermo-Fisher Scientific) and run in MOPS or Tris-Acetate running buffer respectively. Proteins were transferred to PVDF membrane and blocked in 5% milk/TBS-T for 1-2 h. Primary antibodies were diluted in 5% BSA/TBS-T, and incubated overnight at 4°C. Membranes were washed and incubated with anti-rabbit HRP antibody for 1-2 h at room temperature. Membranes were then washed with TBS-T, exposed to chemiluminescent substrate, and imaged using a digital CCD platform (Fluorchem E, Protein Simple, San Jose, CA). Band densitometry was performed using ImageJ software.

### Fluorescence microscopy

Cells were fixed with 4% paraformaldehyde/PBS for 15 min at room temperature, permeabilized with 0.1% Triton X-100/PBS for 10 min at room temperature and blocked with 5% normal goat serum in 0.05% Tween-20/PBS. Cells were then stained with phalloidin-Alexafluor 488 (Thermo Fisher Scientific) for 1 h at room temperature and mounted using medium containing DAPI.

### Flow cytometry

For viability analysis, cells were trypsinized, washed with PBS, and stained with a cell impermeable amine reactive dye (LIVE/DEAD Violet A.K.A. LD405, ThermoFisher Scientific) according to the manufacturer’s instructions. Cells were fixed and permeabilized using the BD Cytofix/Cytoperm kit according to the manufacturer’s instructions (BD Biosciences). Permeabilized cells were stained with fluorophore-conjugated antibodies as indicated in the text. Cells were analyzed on a BD LSR-II cytometer, and data were analyzed using FlowJo software.

## Data availability statement

The original contributions presented in the study are included in the article/**Supplementary Material**. Further inquiries can be directed to the corresponding author.

## Author contributions

CC, FM, JO, NB, AK, and JA have made substantial, direct, and intellectual contributions to the work, and have approved it for publication. All authors contributed to the article and approved the submitted version.

## Acknowledgments

FM was supported by a postdoctoral fellowship of the Canadian Institutes for Health Research. This study was supported by grant R01GM101183 to AK and grants R21AI124266 and P41GM109824 to JA from the National Institutes of Health.

## Conflict of interest

The authors declare that the research was conducted in the absence of any commercial or financial relationships that could be construed as a potential conflict of interest.

## Publisher’s note

All claims expressed in this article are solely those of the authors and do not necessarily represent those of their affiliated organizations, or those of the publisher, the editors and the reviewers. Any product that may be evaluated in this article, or claim that may be made by its manufacturer, is not guaranteed or endorsed by the publisher.

## References

[B1] Arias E. Koga H. Diaz A. Mocholi E. Patel B. Cuervo A. M. (2015). Lysosomal mTORC2/PHLPP1/Akt regulate chaperone-mediated autophagy. Mol. Cell 59, 270–284. doi: 10.1016/j.molcel.2015.05.030 26118642 PMC4506737

[B2] Battaglioni S. Benjamin D. Walchli M. Maier T. Hall M. N. (2022). mTOR substrate phosphorylation in growth control. Cell 185, 1814–1836. doi: 10.1016/j.cell.2022.04.013 35580586

[B3] Benavides-Serrato A. Lee J. Holmes B. Landon K. A. Bashir T. Jung M. E.. (2017). Specific blockade of rictor-mTOR association inhibits mTORC2 activity and is cytotoxic in glioblastoma. PLoS One 12, e0176599. doi: 10.1371/journal.pone.0176599 28453552 PMC5409528

[B4] Buchkovich N. J. Yu Y. Zampieri C. A. Alwine J. C. (2008). The TORrid affairs of viruses: Effects of mammalian DNA viruses on the PI3K-Akt-mTOR signalling pathway. Nat. Rev. Microbiol. 6, 266–275. doi: 10.1038/nrmicro1855 18311165 PMC2597498

[B5] Burnett P. E. Barrow R. K. Cohen N. A. Snyder S. H. Sabatini D. M. (1998). RAFT1 phosphorylation of the translational regulators p70 S6 kinase and 4E-BP1. Proc. Natl. Acad. Sci. U.S.A. 95, 1432–1437. doi: 10.1073/pnas.95.4.1432 9465032 PMC19032

[B6] Carpp L. N. Rogers R. S. Moritz R. L. Aitchison J. D. (2014). Quantitative proteomic analysis of host-virus interactions reveals a role for golgi brefeldin a resistance factor 1 (GBF1) in dengue infection. Mol. Cell Proteomics 13, 2836–2854. doi: 10.1074/mcp.M114.038984 24855065 PMC4223476

[B7] Cristea I. M. Chait B. T. (2011). Conjugation of magnetic beads for immunopurification of protein complexes. Cold Spring Harb. Protoc. 2011, pdbprot5610. doi: 10.1101/pdb.prot5610 PMC6666400 21536766

[B8] Fridy P. C. Li Y. Keegan S. Thompson M. K. Nudelman I. Scheid J. F.. (2014). A robust pipeline for rapid production of versatile nanobody repertoires. Nat. Methods 11, 1253–1260. doi: 10.1038/nmeth.3170 25362362 PMC4272012

[B9] Heaton N. S. Randall G. (2010). Dengue virus-induced autophagy regulates lipid metabolism. Cell Host Microbe 8, 422–432. doi: 10.1016/j.chom.2010.10.006 21075353 PMC3026642

[B10] Jacinto E. Loewith R. Schmidt A. Lin S. Ruegg M. A. Hall A.. (2004). Mammalian TOR complex 2 controls the actin cytoskeleton and is rapamycin insensitive. Nat. Cell Biol. 6, 1122–1128. doi: 10.1038/ncb1183 15467718

[B11] Jordan T. X. Randall G. (2017). Dengue virus activates the AMP kinase-mTOR axis to stimulate a proviral lipophagy. J. Virol. 91(11):e02020-16. doi: 10.1128/JVI.02020-16 28298606 PMC5432877

[B12] Julien L. A. Carriere A. Moreau J. Roux P. P. (2010). mTORC1-activated S6K1 phosphorylates rictor on threonine 1135 and regulates mTORC2 signaling. Mol. Cell Biol. 30, 908–921. doi: 10.1128/MCB.00601-09 19995915 PMC2815569

[B13] Kim L. C. Cook R. S. Chen J. (2017). mTORC1 and mTORC2 in cancer and the tumor microenvironment. Oncogene 36, 2191–2201. doi: 10.1038/onc.2016.363 27748764 PMC5393956

[B14] Kudchodkar S. B. Yu Y. Maguire T. G. Alwine J. C. (2006). Human cytomegalovirus infection alters the substrate specificities and rapamycin sensitivities of raptor- and rictor-containing complexes. Proc. Natl. Acad. Sci. U.S.A. 103, 14182–14187. doi: 10.1073/pnas.0605825103 16959881 PMC1599931

[B15] Kumar A. Buhler S. Selisko B. Davidson A. Mulder K. Canard B.. (2013). Nuclear localization of dengue virus nonstructural protein 5 does not strictly correlate with efficient viral RNA replication and inhibition of type I interferon signaling. J. Virol. 87, 4545–4557. doi: 10.1128/JVI.03083-12 23408610 PMC3624364

[B16] Kuss-Duerkop S. K. Wang J. Mena I. White K. Metreveli G. Sakthivel R.. (2017). Influenza virus differentially activates mTORC1 and mTORC2 signaling to maximize late stage replication. PloS Pathog. 13, e1006635. doi: 10.1371/journal.ppat.1006635 28953980 PMC5617226

[B17] Lahon A. Arya R. P. Banerjea A. C. (2021). Dengue virus dysregulates master transcription factors and PI3K/AKT/mTOR signaling pathway in megakaryocytes. Front. Cell Infect. Microbiol. 11, 715208. doi: 10.3389/fcimb.2021.715208 34513730 PMC8427595

[B18] Lambeth C. R. White L. J. Johnston R. E. de Silva A. M. (2005). Flow cytometry-based assay for titrating dengue virus. J. Clin. Microbiol. 43, 3267–3272. doi: 10.1128/JCM.43.7.3267-3272.2005 16000446 PMC1169137

[B19] Lamming D. W. Demirkan G. Boylan J. M. Mihaylova M. M. Peng T. Ferreira J.. (2014). Hepatic signaling by the mechanistic target of rapamycin complex 2 (mTORC2). FASEB J. 28, 300–315. doi: 10.1096/fj.13-237743 24072782 PMC3868844

[B20] Lampada A. O'Prey J. Szabadkai G. Ryan K. M. Hochhauser D. Salomoni P. (2017). mTORC1-independent autophagy regulates receptor tyrosine kinase phosphorylation in colorectal cancer cells *via* an mTORC2-mediated mechanism. Cell Death Differ. 24, 1045–1062. doi: 10.1038/cdd.2017.41 28475179 PMC5442471

[B21] Laplante M. Sabatini D. M. (2009). mTOR signaling at a glance. J. Cell Sci. 122, 3589–3594. doi: 10.1242/jcs.051011 19812304 PMC2758797

[B22] Laplante M. Sabatini D. M. (2012). mTOR signaling in growth control and disease. Cell 149, 274–293. doi: 10.1016/j.cell.2012.03.017 22500797 PMC3331679

[B23] Lee Y. R. Hu H. Y. Kuo S. H. Lei H. Y. Lin Y. S. Yeh T. M.. (2013). Dengue virus infection induces autophagy: an *in vivo* study. J. BioMed. Sci. 20, 65. doi: 10.1186/1423-0127-20-65 24011333 PMC3848819

[B24] Lee Y. R. Lei H. Y. Liu M. T. Wang J. R. Chen S. H. Jiang-Shieh Y. F.. (2008). Autophagic machinery activated by dengue virus enhances virus replication. Virology 374, 240–248. doi: 10.1016/j.virol.2008.02.016 18353420 PMC7103294

[B25] Le Sage V. Cinti A. Amorim R. Mouland A. J. (2016). Adapting the stress response: Viral subversion of the mTOR signaling pathway. Viruses 8 (6), 152. doi: 10.3390/v8060152 PMC4926172 27231932

[B26] Limonta D. Capo V. Torres G. Perez A. B. Guzman M. G. (2007). Apoptosis in tissues from fatal dengue shock syndrome. J. Clin. Virol. 40, 50–54. doi: 10.1016/j.jcv.2007.04.024 17693133

[B27] Lin J. C. Lin S. C. Chen W. Y. Yen Y. T. Lai C. W. Tao M. H.. (2014). Dengue viral protease interaction with NF-kappaB inhibitor alpha/beta results in endothelial cell apoptosis and hemorrhage development. J. Immunol. 193, 1258–1267. doi: 10.4049/jimmunol.1302675 24973451

[B28] Maiese K. (2020). The mechanistic target of rapamycin (mTOR): Novel considerations as an antiviral treatment. Curr. Neurovasc. Res. 17, 332–337. doi: 10.2174/1567202617666200425205122 32334502 PMC7541431

[B29] Marceau C. D. Puschnik A. S. Majzoub K. Ooi Y. S. Brewer S. M. Fuchs G.. (2016). Genetic dissection of flaviviridae host factors through genome-scale CRISPR screens. Nature 535, 159–163. doi: 10.1038/nature18631 27383987 PMC4964798

[B30] Martins Sde T. Silveira G. F. Alves L. R. Duarte dos Santos C. N. Bordignon J. (2012). Dendritic cell apoptosis and the pathogenesis of dengue. Viruses 4, 2736–2753. doi: 10.3390/v4112736 23202502 PMC3509670

[B31] Mast F. D. Navare A. T. van der Sloot A. M. Coulombe-Huntington J. Rout M. P. Baliga N. S.. (2020). Crippling life support for SARS-CoV-2 and other viruses through synthetic lethality. J. Cell Biol. 219(10), e202006159. doi: 10.1083/jcb.202006159 32785687 PMC7659715

[B32] Mateo R. Nagamine C. M. Spagnolo J. Mendez E. Rahe M. Gale M. Jr.. (2013). Inhibition of cellular autophagy deranges dengue virion maturation. J. Virol. 87, 1312–1321. doi: 10.1128/JVI.02177-12 23175363 PMC3554187

[B33] Metz P. Chiramel A. Chatel-Chaix L. Alvisi G. Bankhead P. Mora-Rodriguez R.. (2015). Dengue virus inhibition of autophagic flux and dependency of viral replication on proteasomal degradation of the autophagy receptor p62. J. Virol. 89, 8026–8041. doi: 10.1128/JVI.00787-15 26018155 PMC4505648

[B34] Oh W. J. Jacinto E. (2011). mTOR complex 2 signaling and functions. Cell Cycle 10, 2305–2316. doi: 10.4161/cc.10.14.16586 21670596 PMC3322468

[B35] Orzalli M. H. Kagan J. C. (2017). Apoptosis and necroptosis as host defense strategies to prevent viral infection. Trends Cell Biol. 27, 800–809. doi: 10.1016/j.tcb.2017.05.007 28642032 PMC5653411

[B36] Pascual J. Royuela A. Fernandez A. M. Herrero I. Delgado J. F. Sole A.. (2016). Role of mTOR inhibitors for the control of viral infection in solid organ transplant recipients. Transpl. Infect. Dis. 18, 819–831. doi: 10.1111/tid.12601 27600985

[B37] Pryor M. J. Carr J. M. Hocking H. Davidson A. D. Li P. Wright P. J. (2001). Replication of dengue virus type 2 in human monocyte-derived macrophages: comparisons of isolates and recombinant viruses with substitutions at amino acid 390 in the envelope glycoprotein. Am. J. Trop. Med. Hyg. 65, 427–434. doi: 10.4269/ajtmh.2001.65.427 11716094

[B38] Sarbassov D. D. Ali S. M. Kim D. H. Guertin D. A. Latek R. R. Erdjument-Bromage H.. (2004). Rictor, a novel binding partner of mTOR, defines a rapamycin-insensitive and raptor-independent pathway that regulates the cytoskeleton. Curr. Biol. 14, 1296–1302. doi: 10.1016/j.cub.2004.06.054 15268862

[B39] Sarbassov D. D. Guertin D. A. Ali S. M. Sabatini D. M. (2005). Phosphorylation and regulation of Akt/PKB by the rictor-mTOR complex. Science 307, 1098–1101. doi: 10.1126/science.1106148 15718470

[B40] Savidis G. McDougall W. M. Meraner P. Perreira J. M. Portmann J. M. Trincucci G.. (2016). Identification of zika virus and dengue virus dependency factors using functional genomics. Cell Rep. 16, 232–246. doi: 10.1016/j.celrep.2016.06.028 27342126

[B41] Sessions O. M. Barrows N. J. Souza-Neto J. A. Robinson T. J. Hershey C. L. Rodgers M. A.. (2009). Discovery of insect and human dengue virus host factors. Nature 458, 1047–1050. doi: 10.1038/nature07967 19396146 PMC3462662

[B42] Shives K. D. Beatman E. L. Chamanian M. O'Brien C. Hobson-Peters J. Beckham J. D. (2014). West Nile virus-induced activation of mammalian target of rapamycin complex 1 supports viral growth and viral protein expression. J. Virol. 88, 9458–9471. doi: 10.1128/JVI.01323-14 24920798 PMC4136264

[B43] Simcox J. Lamming D. W. (2022). The central moTOR of metabolism. Dev. Cell 57, 691–706. doi: 10.1016/j.devcel.2022.02.024 35316619 PMC9004513

[B44] Suksanpaisan L. Cabrera-Hernandez A. Smith D. R. (2007). Infection of human primary hepatocytes with dengue virus serotype 2. J. Med. Virol. 79, 300–307. doi: 10.1002/jmv.20798 17245728

[B45] Tackett A. J. DeGrasse J. A. Sekedat M. D. Oeffinger M. Rout M. P. Chait B. T. (2005). I-DIRT, a general method for distinguishing between specific and nonspecific protein interactions. J. Proteome Res. 4, 1752–1756. doi: 10.1021/pr050225e 16212429

[B46] Thepparit C. Khakpoor A. Khongwichit S. Wikan N. Fongsaran C. Chingsuwanrote P.. (2013). Dengue 2 infection of HepG2 liver cells results in endoplasmic reticulum stress and induction of multiple pathways of cell death. BMC Res. Notes 6, 372. doi: 10.1186/1756-0500-6-372 24034452 PMC3847886

[B47] Torrentes-Carvalho A. Azeredo E. L. Reis S. R. Miranda A. S. Gandini M. Barbosa L. S.. (2009). Dengue-2 infection and the induction of apoptosis in human primary monocytes. Mem. Inst. Oswaldo Cruz 104, 1091–1099. doi: 10.1590/S0074-02762009000800005s 20140369

[B48] Tyakht A. V. Ilina E. N. Alexeev D. G. Ischenko D. S. Gorbachev A. Y. Semashko T. A.. (2014). RNA-Seq gene expression profiling of HepG2 cells: The influence of experimental factors and comparison with liver tissue. BMC Genomics 15, 1108. doi: 10.1186/1471-2164-15-1108 25511409 PMC4378340

[B49] Xie J. Wang X. Proud C. G. (2016). mTOR inhibitors in cancer therapy. 5, 2078. doi: 10.12688/f1000research.9207.1 PMC5007757 27635236

[B50] Zinzalla V. Stracka D. Oppliger W. Hall M. N. (2011). Activation of mTORC2 by association with the ribosome. Cell 144, 757–768. doi: 10.1016/j.cell.2011.02.014 21376236

[B51] Zou Z. Chen J. Yang J. Bai X. (2016). Targeted inhibition of Rictor/mTORC2 in cancer treatment: A new era after rapamycin. Curr. Cancer Drug Targets 16, 288–304. doi: 10.2174/1568009616666151113120830 26563881

