# Supplementary material for: Dengue activates mTORC2 signaling to counteract apoptosis and maximize viral replication
Source: Front Cell Infect Microbiol. 2022 Sep 12;12:979996. doi: 10.3389/fcimb.2022.979996 (PMC9510660; doi:10.3389/fcimb.2022.979996)
Supplement: Supplementary file 1 [file DataSheet_1.docx]

**Carter et al., 2022 Supplemental Figure 1**

**
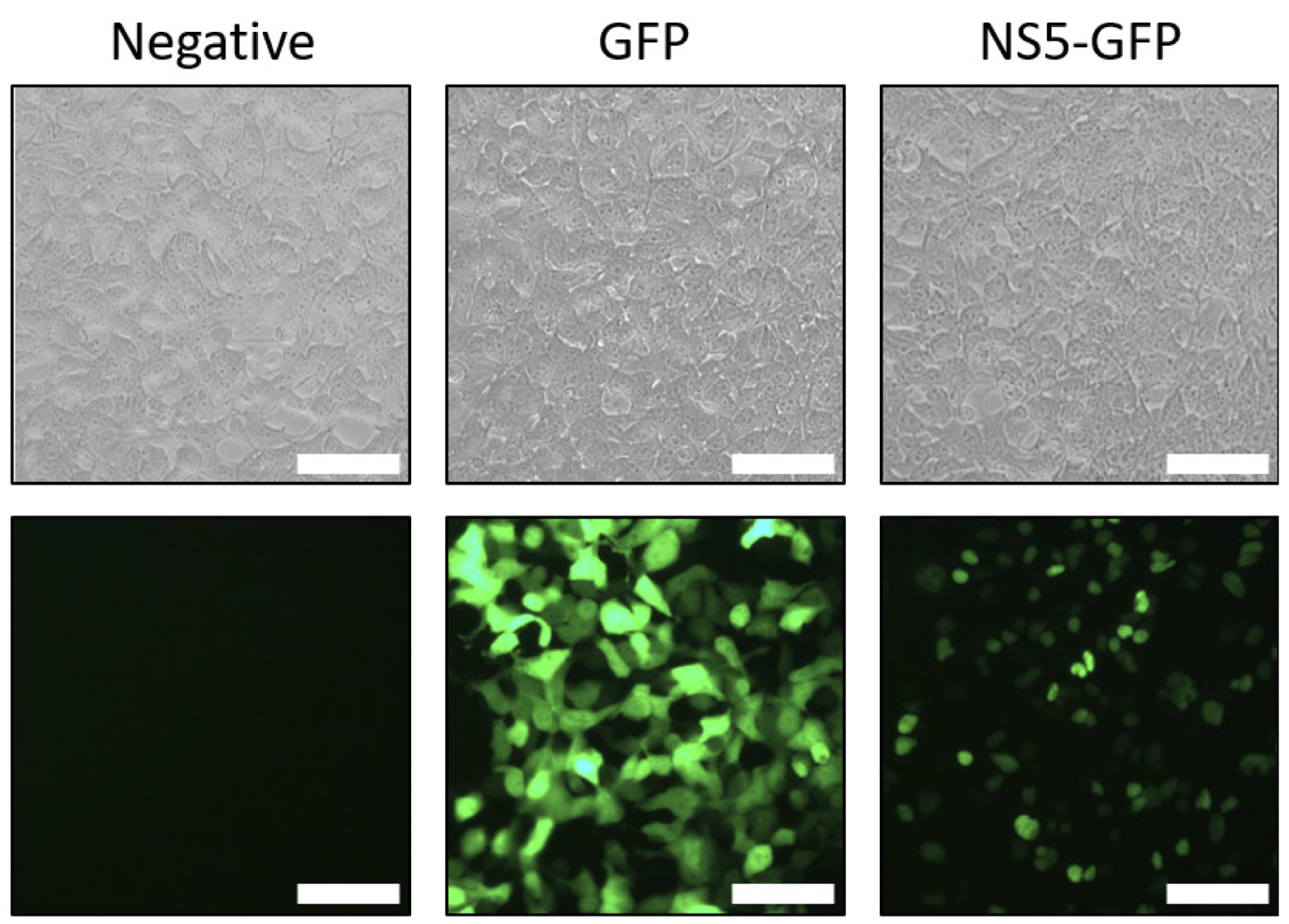
**

SUPPLEMENTAL FIGURE 1**. Expression of NS5-GFP fusion protein.** 293FT cells were transfected with expression plasmids encoding GFP or NS5-GFP fusion protein, and live cells were imaged by fluorescence microscopy. Bar = 100 µm.
